# Magnetic Mesoporous Calcium Sillicate/Chitosan Porous Scaffolds for Enhanced Bone Regeneration and Photothermal-Chemotherapy of Osteosarcoma

**DOI:** 10.1038/s41598-018-25595-2

**Published:** 2018-05-09

**Authors:** Fan Yang, Jiawei Lu, Qinfei Ke, Xiaoyuan Peng, Yaping Guo, Xuetao Xie

**Affiliations:** 1 Department of Orthopedic Surgery, Shanghai Jiao Tong University Affiliated Sixth People’s Hospital, Shanghai, China; 2 The Education Ministry Key Lab of Resource Chemistry and Shanghai Key Laboratory of Rare Earth Functional Materials, Shanghai Normal University, Shanghai, China

## Abstract

The development of multifunctional biomaterials to repair bone defects after neoplasm removal and inhibit tumor recurrence remained huge clinical challenges. Here, we demonstrate a kind of innovative and multifunctional magnetic mesoporous calcium sillicate/chitosan (MCSC) porous scaffolds, made of M-type ferrite particles (SrFe_12_O_19_), mesoporous calcium silicate (CaSiO_3_) and chitosan (CS), which exert robust anti-tumor and bone regeneration properties. The mesopores in the CaSiO_3_ microspheres contributed to the drug delivery property, and the SrFe_12_O_19_ particles improved photothermal therapy (PTT) conversion efficacy. With the irradiation of NIR laser, doxorubicin (DOX) was rapidly released from the MCSC/DOX scaffolds. *In vitro* and *in vivo* tests demonstrated that the MCSC scaffolds possessed the excellent anti-tumor efficacy via the synergetic effect of DOX drug release and hyperthermia ablation. Moreover, BMP-2/Smad/Runx2 pathway was involved in the MCSC scaffolds promoted proliferation and osteogenic differentiation of human bone marrow stromal cells (hBMSCs). Taken together, the MCSC scaffolds have the ability to promote osteogenesis and enhance synergetic photothermal-chemotherapy against osteosarcoma, indicating MCSC scaffolds may have great application potential for bone tumor-related defects.

## Introduction

Bone metastasis has been commonly observed in malignant tumors, notably for patients with breast cancer, lung cancer or kidney cancer^1,2^. The conventional therapeutic strategies for bone tumors include surgical intervention and chemo/radiotherapy, but these approaches often fail to eradicate residual malignant cells, which confer the potential for recurrence^3^. Additionally, bone defect affects the quality of life in patients receiving surgical resection; chemo/radiotherapy may cause side effects and drug resistance^4^. Previous studies suggested that residual tumor cells could be effectively killed by controlled drug delivery system mediated photothermal therapy (PTT)^5,6^. Local drug delivery systems could facilitate the release of anti-cancer drugs at designated sites with higher local drug concentrations, and minimize the cytotoxicity to normal cells^7^. Mesoporous CaSiO_3_ has been widely used for both controlled drug delivery systems and bone repair applications due to good biocompatibility, drug loading efficiency and sustained drug release performance^8^. The chemotherapeutic drugs loaded-mesoporous CaSiO_3_ scaffolds may combine bone regenerative abilities with anti-tumor properties. Nevertheless, multifunctional biomaterials with optimal anti-tumor and bone regeneration properties are rarely reported.

PTT has been shown to be an effective, non-invasive and low cytotoxicity strategy to kill tumor cells^9–11^. Conventional photothermal agents mainly include gold nanomaterials^12,13^, copper nanomaterials^14^, carbonnano materials^15^, near infrared (NIR) dyes^16,17^ and magnetic ironoxide nanoparticles^18,19^, in which these regimens show good NIR absorption property. Compared with the conventional photothermal agents, the magnetic iron oxide particles exhibited higher NIR absorbance, higher photothermal-conversion efficiency, better thermal conductivity and cytocompatibility^18,19^. The NIR irradiation could elevate local temperatures of photothermal particles up to 42~50 °C, thus facilitating tumor hyperthermia ablation^20^. Moreover, the photothermal treatment can trigger the rapid release of chemotherapeutic drugs from the scaffolds^21^, and promote cell membrane permeability of drug incorporation^22^. Therefore, it could be inferred that the photothermal agents could synergize with chemotherapy to drive potent anti-tumor responses for malignant cells.

The commonly utilized bone repair materials, including hydroxyapatite (HA), CaSiO_3_, bioglass (BG), poly (methyl methacrylate) (PMMA) and chitosan (CS), possess desirable osteoconductivity, but their osteoinductivity is insufficient^23,24^. Previous study reported that the use of static magnetic fields (SMF) could stimulate osteogenic differentiation of human bone marrow-derived mesenchymal stem cells (hBMSCs) *in vitro* and initiate early bone formation *in vivo* as indicated by the upregulation of osteogenic markers, such as alkaline phosphatase (ALP), runt-related transcription factor 2 (Runx2), collagen1a1 (COL1a1), osteocalcin (OCN), osteonectin (ON), osteopontin (OPN), and osterix (OSX)^25^. In addition, magnetic nanoparticles loaded biopolymer scaffolds promoted osteoblastic cells adhesion and differentiation and bone formation *in vivo*^26^. However, the current magnetic iron oxide nanoparticles (such as Fe_3_O_4_) exhibit too weak magnetism to represent remarkable osteogenesis effect. Therefore, the scaffold incorporated with M-type ferrite particles may overcome these drawbacks, and may be considered a promising biomatrix for bone engineering.

**Figure 1:**
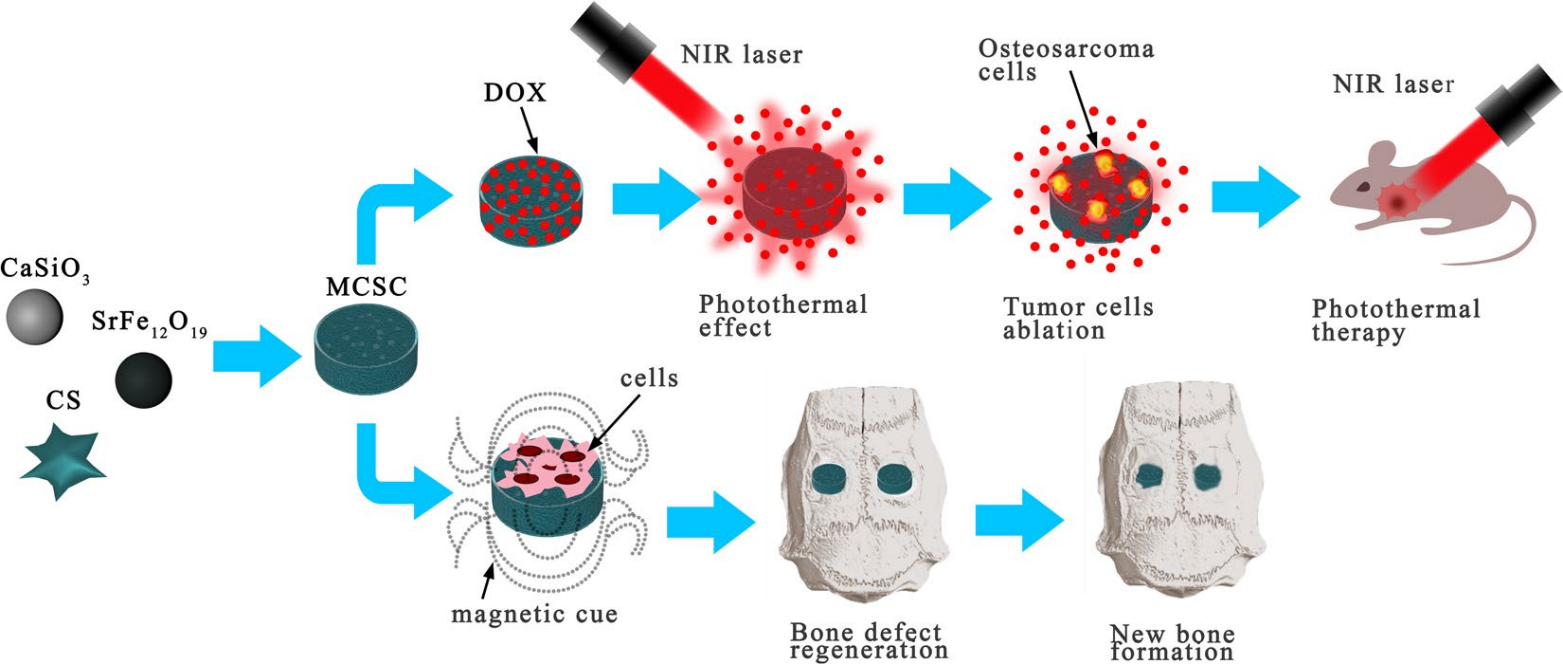
The graphic abstract of the present study. The dispersing of M-type ferrite particles and mesoporous calcium silicate microspheres within chitosan films consist MCSC scaffolds. The MCSC scaffolds exhibit excellent property in drug delivery and simultaneously improve the efficacy of photothermal therapy under the irradiation of NIR laser. Additionally, the MCSC scaffolds also enhance new bone regeneration effectively by promoting osteogenic differentiation. NIR, near infrared.

Calcium sillicate and chitosan have been widely used for bone filling materials or bone scaffolds for their good biocompatibility and bioactivity^27,28^. SrFe_12_O_19_ is one of the M-type ferrite materials showing strong intrinsic magnetic potential^29^. Here, we introduced a novel multifunctional magnetic mesoporous calcium sillicate/chitosan porous scaffold, which is made of SrFe_12_O_19_, CaSiO_3_ and CS, possessing both potent anti-tumor and osteogenesis regeneration properties (Fig. 1).

## Results

Ethical approval for this investigation was obtained from the Research Ethics Committee of the Shanghai Sixth People’s Hospital-affiliated Shanghai Jiao Tong University. All methods were carried out in accordance with relevant guidelines and regulations of the Research Ethics Committee of the Shanghai Sixth People’s Hospital-affiliated Shanghai Jiao Tong University, all experimental protocols were approved by the Research Ethics Committee of the Shanghai Sixth People’s Hospital-affiliated Shanghai Jiao Tong University. Research carried out on humans must be in compliance with the Helsinki Declaration, human bone marrow-derived mesenchymal stem cells (hBMSCs) and bone tissue were obtained from four donors who gave their written informed consent.

### Preparation and characterization of the scaffolds

#### Morphology, mesoporous structure of calcium sillicate microspheres

**Figure 2:**
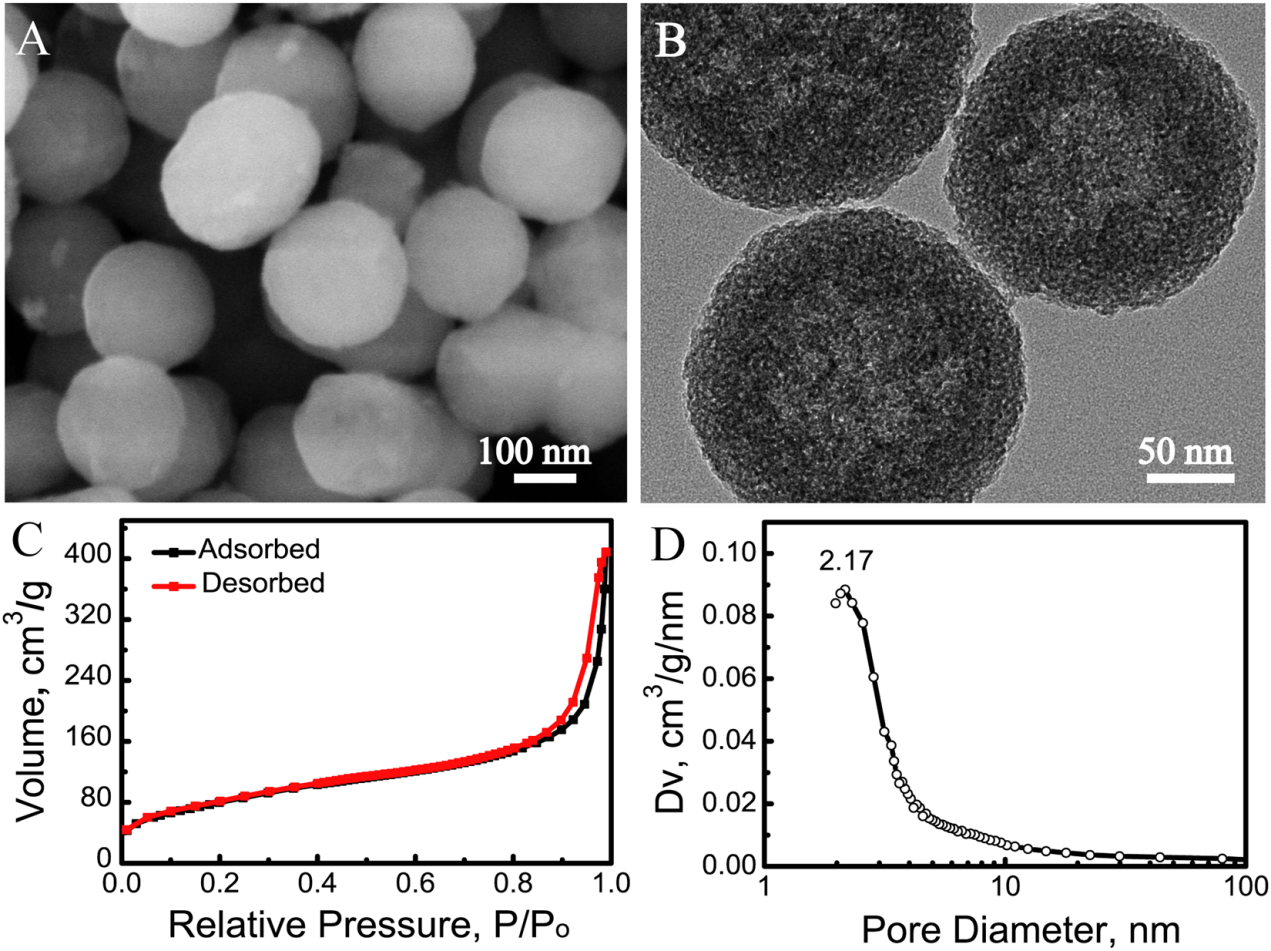
Characterization of scaffolds. (**A**) SEM image, (**B**) TEM image, (**C**) Nitrogen adsorption-desorption isotherm and (**D**) Pore size distribution curve of mesoporous CaSiO_3_ microspheres. SEM, emission scanning electron microscopy; TEM, transmission electron microscopy.

Mesoporous calcium sillicate microspheres were prepared by using cetyltrimethyl ammonium bromide (CTAB). The emission scanning electron microscopy (SEM) analysis revealed that the sizes of microspheres were around 200 nm (Fig. 2A). Additionally, the light-shaded spots within the microspheres could be detected by transmission electron microscopy (TEM) (Fig. 2B), which suggest the mesoporous structure. As depicted in Fig. 2C, the nitrogen adsorption-desorption isotherms of calcium silicate microspheres had the type IV isotherms with type H3 hysteresis loops. Moreover, no limiting adsorption at high P/Po in the type H3 loop demonstrated that the mesopores within the microspheres exhibited the slit-shaped pores with pore size of approximately 2.17 nm (Fig. 2D), which was consistent with the result of TEM analysis. The mesoporous structure significantly increased the BET surface area and pore volume of calcium silicate microspheres up to 291.57 m^2^/g and 0.41 cm^3^/g, respectively (Fig. 2D).

#### Morphology of magnetic mesoporous calcium sillicate/chitosan scaffolds

**Figure 3:**
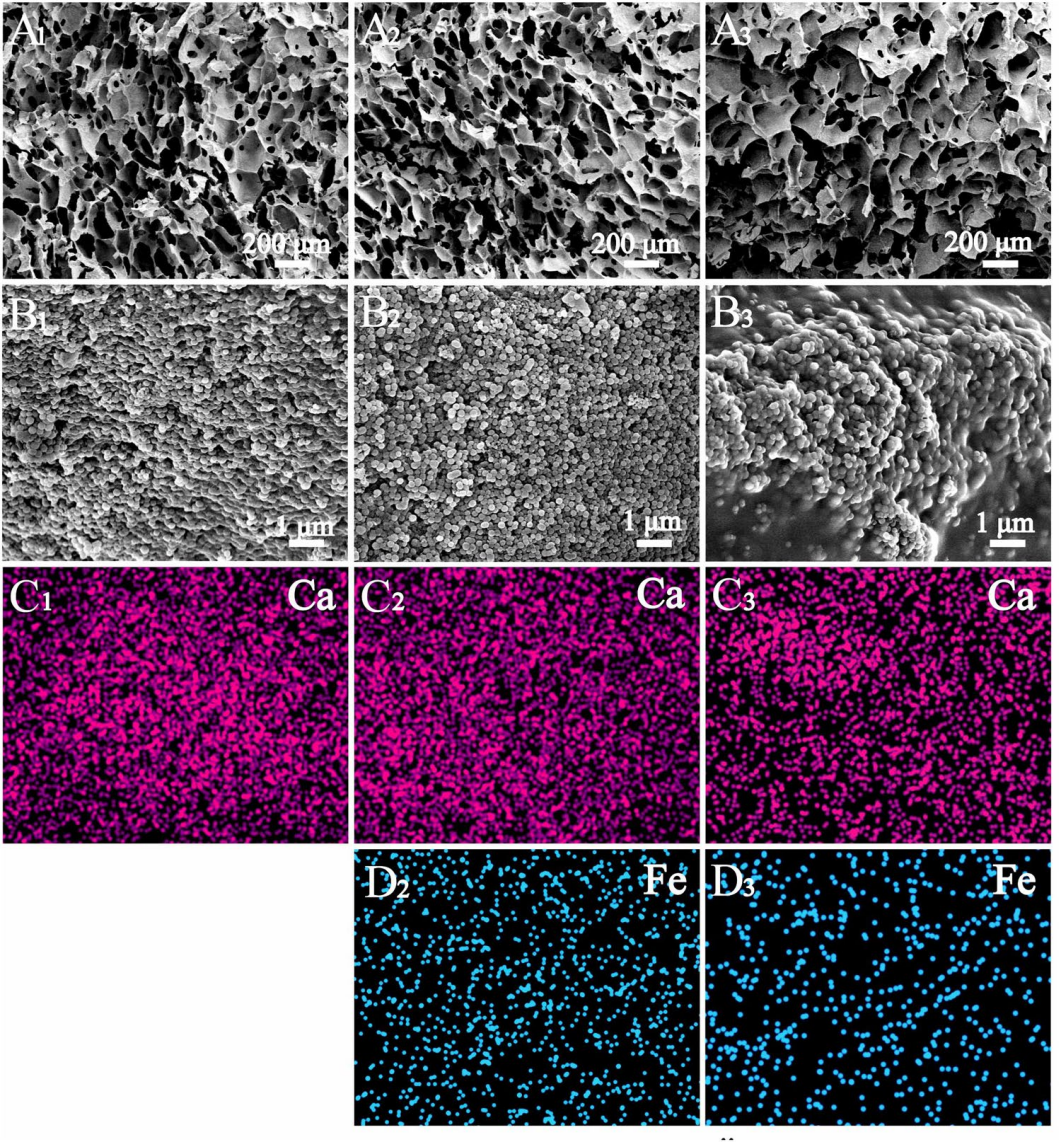
The key elements distribution of on scaffolds evaluated by SEM. Ca and Fe element distribution images of samples: (A1, B1, C1) CSC scaffolds, (A2, B2, C2, D2) MCSC 1:7 scaffolds and (A3, B3, C3, D3) MCSC 1:3 scaffolds. SEM, emission scanning electron microscopy.

Next the morphology, structure and magnetic property of multifunctional magnetic mesoporous calcium sillicate/chitosan (MCSC) scaffolds were assessed. The MCSC scaffolds with the ferrite/calcium silicate mass ratio at 1:7 and 1:3 were named as MCSC 1:7 and MCSC 1:3, respectively. The three-dimensional interconnected macroporous structure of CSC (Fig. 3A_1_), MCSC 1:7 (Fig. 3A_2_) and MCSC 1:3 (Fig. 3A_1_) scaffolds were assessed which showed pore sizes ranging from 100 to 300 μm. As shown in Fig. 3C_1–3_, the mesoporous CaSiO_3_ microspheres and iron particles were uniformly distributed within and/or on CS films. The calcium (Ca) was derived from the CaSiO_3_ microspheres (Fig. 3C_1–3_), and the iron (Fe) was released by SrFe_12_O_19_ (Fig. 3C_2–3_).

#### Phase structures and property of magnetic mesoporous calcium sillicate/chitosan scaffolds

**Figure 4:**
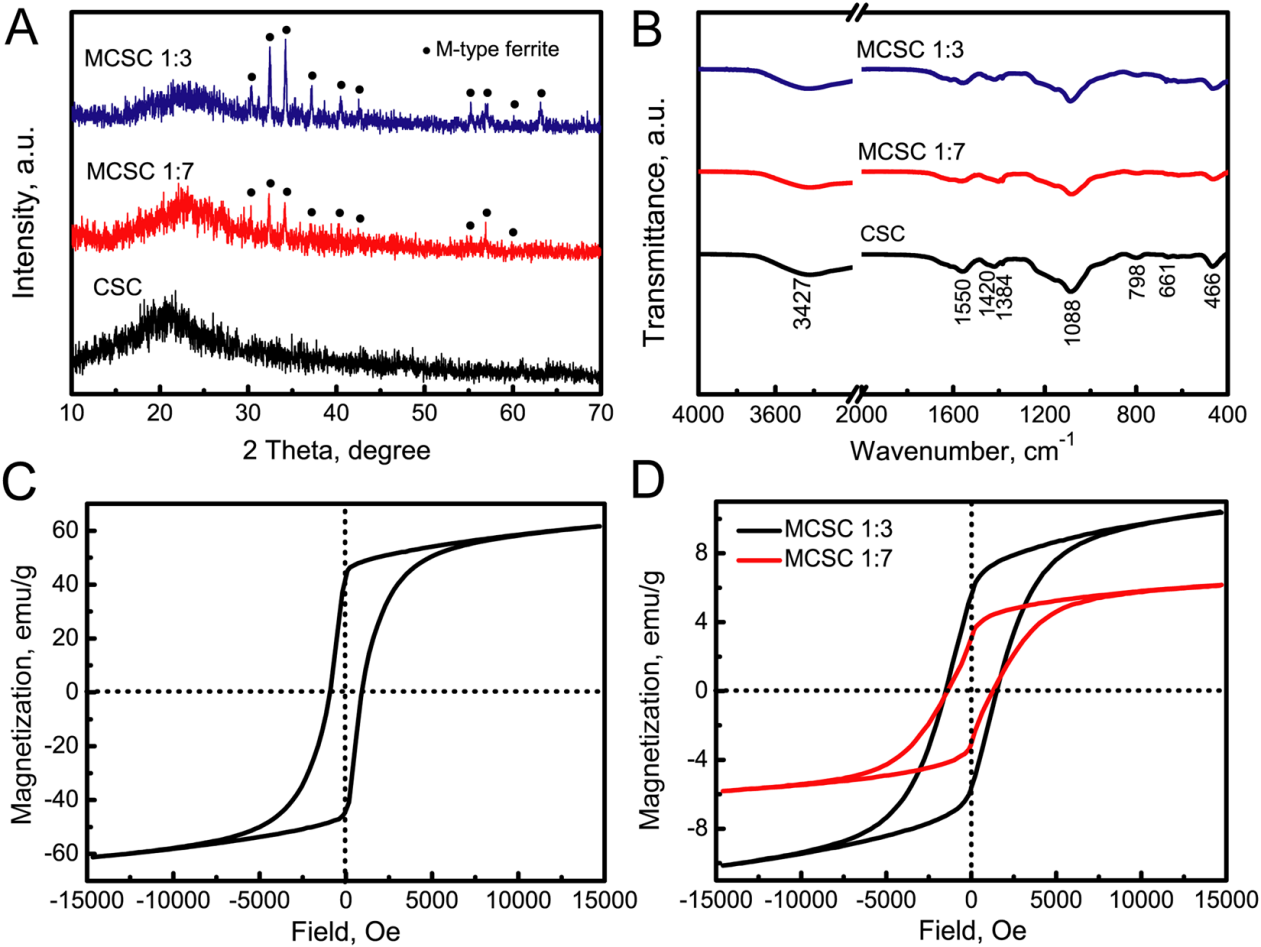
The phases of scaffolds were characterized by XRD and FTIR. (**A**) XRD patterns and (**B**) FTIR spectra of CSC, MCSC 1:7, MCSC 1:3 scaffolds; magnetic hysteresis loops of (**C**) pure magnetic particles, (**D**) MCSC 1:3 and MCSC 1:7 scaffolds. XRD, X-ray power diffraction; FTIR, fourier transform infrared.

The crystalline structure of CSC, MCSC 1:7 and MCSC 1:3 scaffolds were characterized by X-ray power diffraction (XRD) assay. As the CaSiO_3_ was non-crystalline and CS was semi-crystalline kind of material, therefore, only a broad peak at 2*θ* = 22° was detected for the CSC porous scaffolds (Fig. 4A). After the incorporation of magnetic particles in the CSC scaffolds, the characteristic peaks due to M-type ferrite were observed for both the MCSC 1:7 and MCSC 1:3 scaffolds. With the increment ratio of magnetic particles, the peak strengths were enhanced at corresponding points (Fig. 4A). Then, fourier transform infrared (FTIR) spectra was used to characterize the functional groups of the CSC, MCSC 1:7 and MCSC 1:3 scaffolds. As shown in Fig. 4B, all these three scaffolds had comparable adsorption peaks. Then, the magnetic property measurement of MCSC scaffolds was evaluated. The saturated magnetization (Ms) and coercivity (Hc) of M-type strontium hexagonal ferrites (SrFe_12_O_19_) was 61.66 emu/g and 919 Oe, respectively (Fig. 4C). After the ferrite particles were incorporated in scaffolds, the MCSC 1:7 and MCSC 1:3 scaffolds both exhibited good magnetic property (Fig. 4D). The saturated magnetization value of the MCSC 1:3 scaffolds (10.36 emu·g^−1^) were greater than the MCSC 1:7 scaffolds (6.10 emu/g), indicating the ratio of ferrite particles in the scaffolds was positively related to magnetization (Fig. 4D). Moreover, both the MCSC 1:7 and MCSC 1:3 scaffolds exhibited similar coercivities (Hc) (1279 and 1510 Oe, respectively) (Fig. 4D). The higher saturation magnetization and coercivity of MCSC scaffolds resulted in the high magnetic field strength.

#### NIR photothermal conversion efficiency of magnetic mesoporous calcium sillicate/chitosan scaffolds

**Figure 5:**
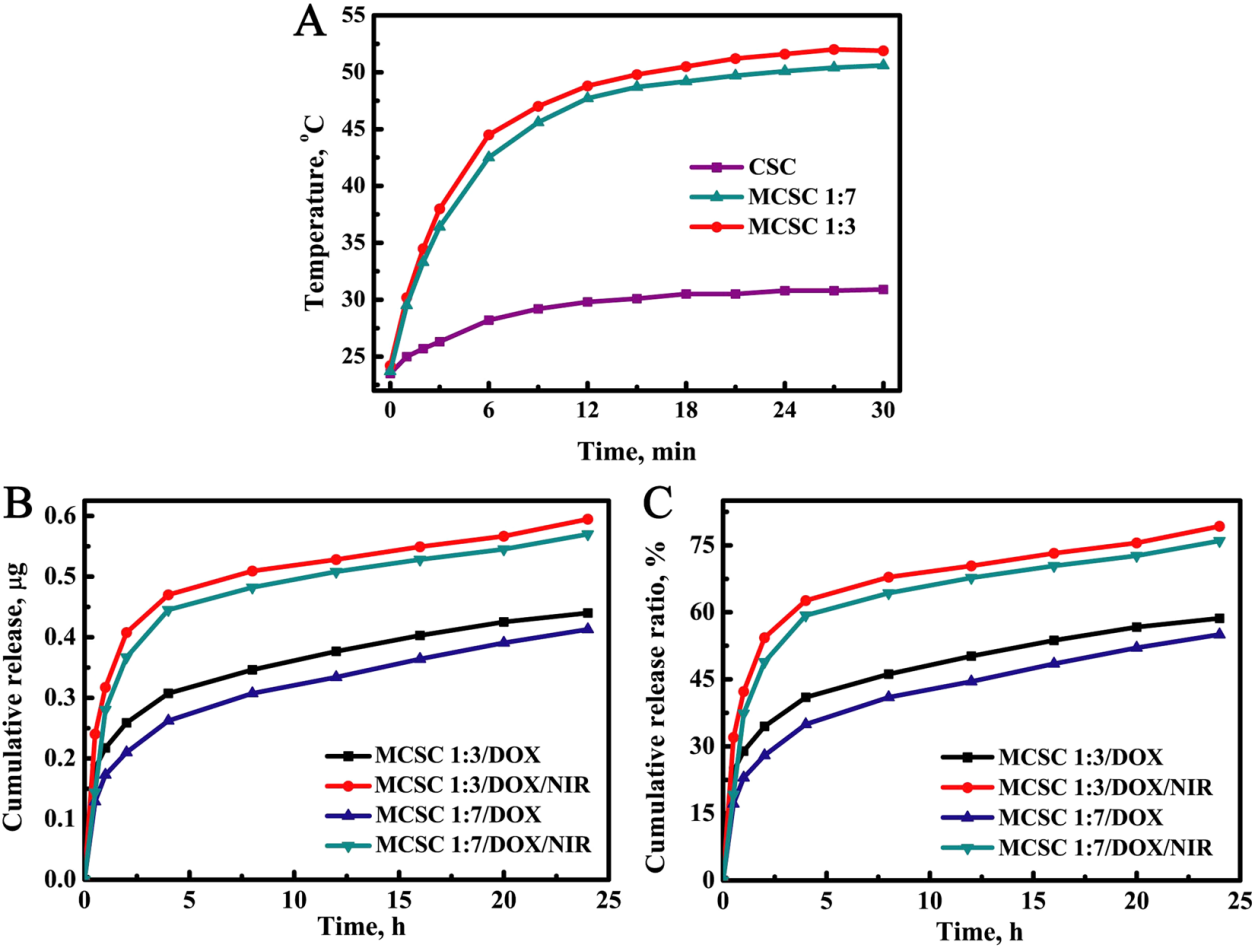
Photothermal conversion efficiency and drug release profiles of scaffolds. (**A**) Temperatures changes post the irradiation of NIR laser for CSC, MCSC 1:7 and MCSC 1:3 scaffolds. (**B**) *In vitro* drug cumulative release amounts and (**C**) cumulative release ratios from MCSC 1:7/DOX and MCSC 1:3/DOX scaffolds in the presence or absence of NIR irradiation. NIR, near infrared.

Compared with CSC scaffolds, the MCSC scaffolds possessed better photothermal conversion efficiency (Fig. 5A). With the irradiation of NIR laser, the temperature in both the MCSC 1:7 and MCSC 1:3 scaffolds in mediums was gradually increased (Fig. 5A). In addition to PTT, controlled drug delivery therapy is an effective approach to kill malignant cells. Doxorubicin (DOX), a widely used chemotherapy medication, was employed to evaluate the efficacy of MCSC as a drug carrier. The drug release profiles showed that DOX was gradually released from its carriers and the MCSC 1:7/DOX scaffolds had a similar drug release profile to that of the MCSC 1:3/DOX scaffolds (Fig. 5B,C). Notably, the NIR laser irradiation accelerated the DOX release ratio and the drug cumulative release ratio at 24 h for the MCSC 1:7/DOX, MCSC 1:3/DOX, MCSC 1:7/DOX/NIR and MCSC 1:3/DOX/NIR was 55.0%, 58.6%, 76.0% and 79.3%, respectively (Fig. 5B,C). The rapid release of DOX from MCSC scaffolds may have the potential to reduce systemic cytotoxicity for the higher local drug concentrations in tumors.

### Application of the MCSC scaffolds in photothermal and anti-cancer therapy

#### *In vitro* analyses for anti-tumor effect

**Figure 6:**
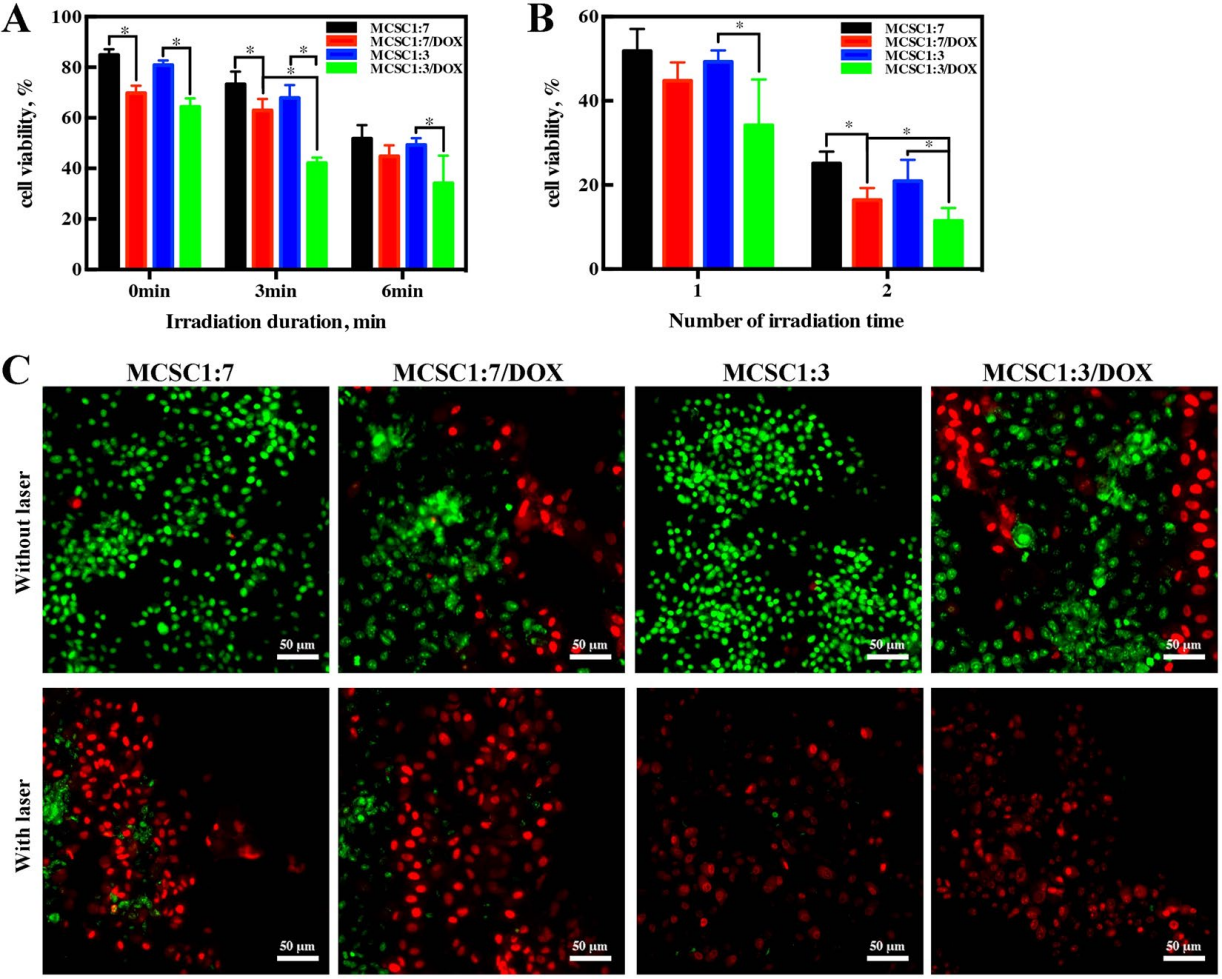
The scaffolds exert potent anti-proliferative effects on cancer cells *in vitro*. MG-63 cell viability post MCSC 1:7, MCSC 1:7/DOX, MCSC 1:3 and MCSC 1:3/DOX scaffolds treatment for 48 h with or without NIR irradiation (**A**) and indicated NIR irradiation times (**B**). (**C**) Cell viability based on Live/Dead assays, green for live cells and red for dead cells (all scale bars = 50 μm). *P < 0.05.

In order to evaluate the synergism of MCSC scaffolds combined with PTT in combating tumor proliferation, their anti-tumor effects were tested both *in vitro* and *in vivo*. After incubation with MCSC 1:7 and MCSC 1:3 scaffolds for 24 h, MG-63 cells exhibited high cell viability indicating that both MCSC 1:7 and MCSC 1:3 scaffolds had good biocompatibility (Fig. 6A). MCSC 1:7/DOX and MCSC 1:3/DOX scaffolds treatment showed moderate anti-proliferative impacts on MG-63 cells (Fig. 6A). Next, we examined the synergy between MCSC scaffolds and PTT. MG-63 cells were incubated with MCSC scaffolds for 24 h, followed by exposure to laser illumination. As expected, laser irradiation combined with MCSC scaffolds exhibit potent anti-proliferative effects on MG-63 cells in a dose dependent manner, and laser irradiation enhanced the anti-tumor response of MCSC/DOX scaffolds as indicated by lower cell viability compared with cells treated with MCSC/DOX alone (Fig. 6A). Among them, the proliferative arrest effect in MCSC 1:7/DOX and MCSC 1:3/DOX scaffolds was stronger than those of MCSC 1:7 and MCSC 1:3 scaffolds when exposed to laser irradiation (Fig. 6A). Furthermore, cells irradiated twice exhibited a more significant proliferative arrest response and the MCSC 1:3/DOX scaffolds had a more profound cytotoxicity effect than that of MCSC 1:7/DOX scaffolds (Fig. 6B). The live/dead assay was used to validate the phenomenon. In consistent with previous findings, the MCSC 1:7/DOX and MCSC 1:3/DOX scaffolds had moderate cytotoxic effect on MG-63 cells. When cells was exposed to NIR, the MCSC scaffolds showed strong cytotoxic effect on MG-63 cells as almost no green signals was detected in MCSC 1:3 and MCSC 1:3/DOX treated groups (Fig. 6C).

#### *In vivo* assay for anti-tumor effects

**Figure 7:**
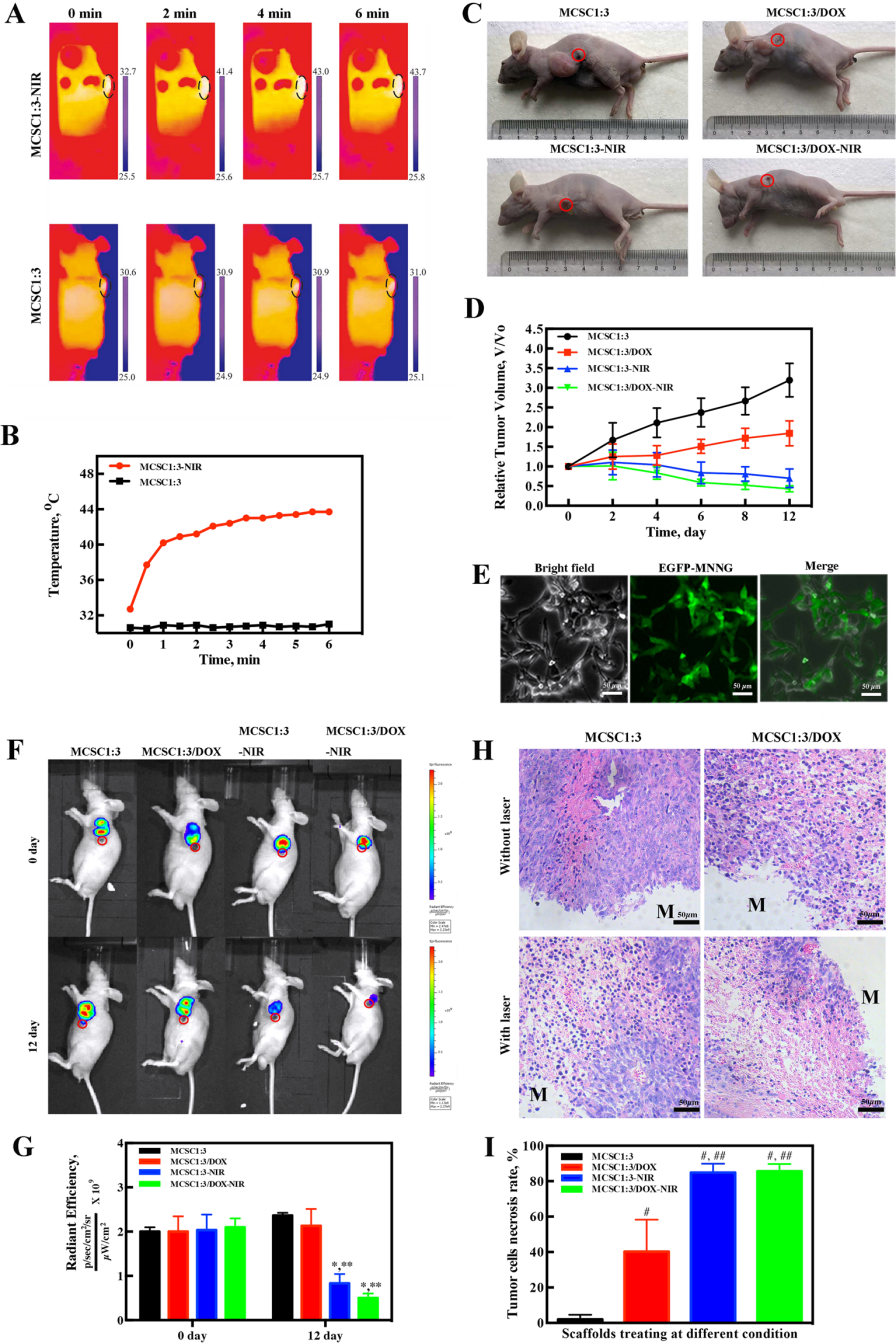
The MCSC scaffolds exhibit robust anti-tumor effects *in vivo*. (**A**) IR thermal images and (**B**) temperature curve of MNNG xenograft mice exposed to NIR laser irradiation (red) or not (black). (**C**) Representative images for tumor volumes at day 12. (**D**) Relative tumor volume changes. (**E**) Lentivirus containing EGFP were successfully transfected in MNNG as indicated by fluorescence. (**F**) Representative images for the whole-body fluorescence imaging of tumors and (**G**) statistical analysis for intensity and area at day 0 and day 12. (**H**) Representative histology images of tumor tissues, M represents implanted scaffolds material. (**I**) Statistical analysis of tumor cell necrosis rate. *P < 0.05. IR, infrared.

To further understand the synergistic effect of PTT in combination with MCSC 1:3 or MCSC 1:3/DOX scaffolds on anti-tumor effects, *in vivo* analyses were conducted and MNNG xenograft mouse model was established. Upon the NIR irradiation, the temperature in the tumor loci injected with the MCSC 1:3 scaffolds increased to approximately 44 °C (Fig. 7A). However, the temperature was comparable before and after the treatment of MCSC 1:3 scaffolds alone around the tumor loci (Fig. 7A,B). Next, the anti-tumor effects of MCSC 1:3 and MCSC 1:3/DOX scaffolds were evaluated. Compared with MCSC 1:3 scaffolds, MCSC 1:3/DOX scaffolds significantly inhibited tumor proliferation, indicating that MCSC 1:3/DOX scaffolds had anti-tumor responses *in vivo* (Fig. 7C,D). After NIR laser irradiation, the tumor volumes of MCSC 1:3-NIR mice and MCSC 1:3/DOX-NIR mice were significantly decreased (Fig. 7D). The tumor volumes in MCSC 1:3/DOX-NIR mice were the smallest among others (Fig. 7C,D). Furthermore, MNNG cells were transfected with lentivirus containing enhanced green fluorescent protein genes (EGFP) (Fig. 7E) and again xenograft mouse model was established. Compared to day 0, the tumor volume was increased in mice treated with MCSC 1:3 and remained comparable in mice treated with MCSC1:3/DOX (Fig. 7F,G). In contrast, both the fluorescence intensity and area were significantly reduced in mice treated with the MCSC 1:3-NIR and MCSC1:3/DOX-NIR at day 12 and mice treated with MCSC1:3/DOX-NIR showed a more remarkable decrease (Fig. 7F,G). Hematoxylin and eosin (H&E) staining revealed that the MCSC 1:3-NIR and MCSC 1:3/DOX-NIR induced significantly higher cell necrosis ratio compared with MCSC 1:3 and MCSC 1:3/DOX scaffolds (Fig. 7H,I). These findings indicate that PTT could synergize with MCSC to achieve potent anti-tumor effects both *in vitro* and *in vivo*.

### Application of the scaffolds in bone tissue regeneration

#### The evaluation of MCSC scaffolds in bone regeneration *in vitro*

**Figure 8:**
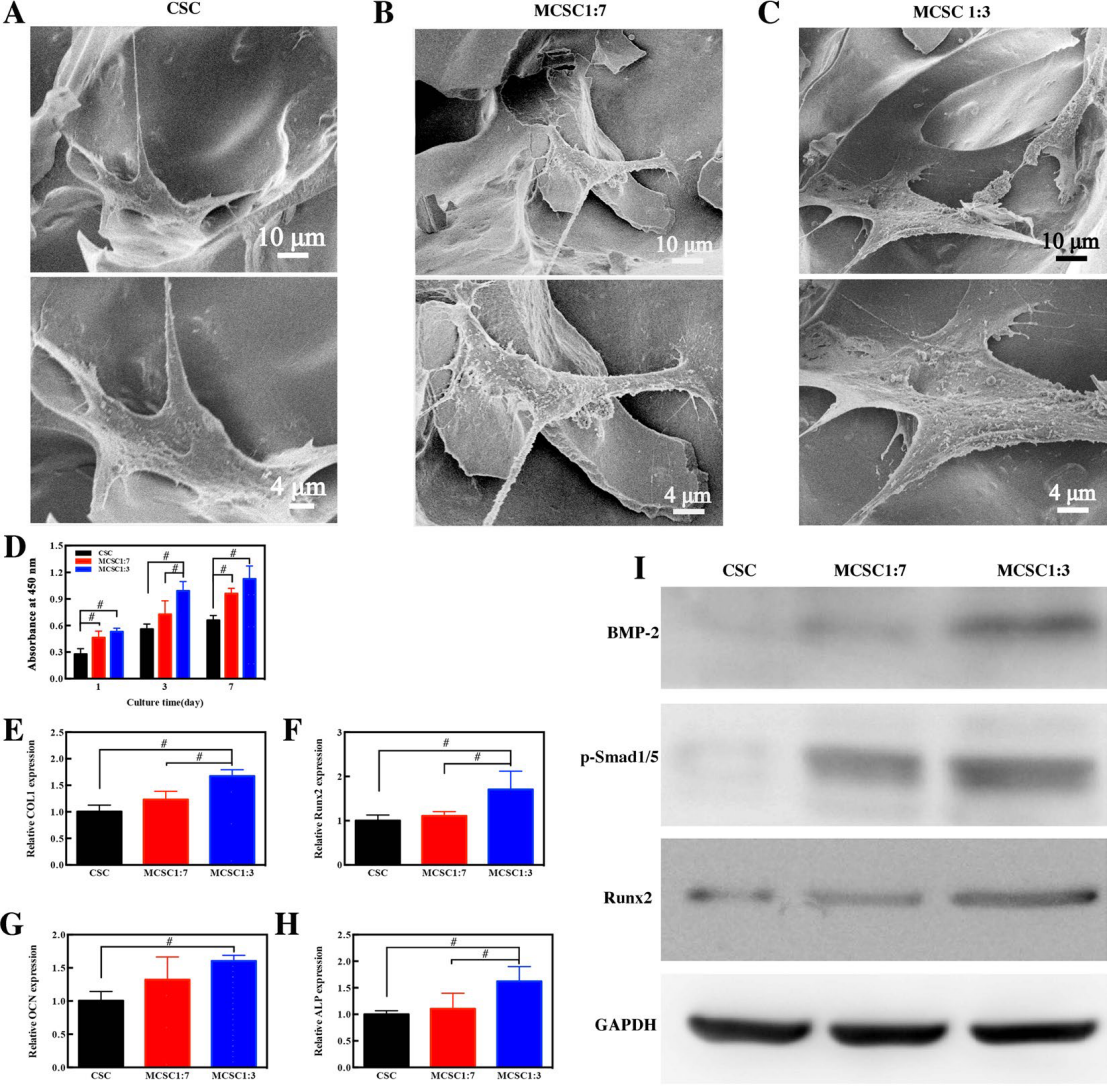
The hBMSCs seeded scaffolds promoted osteogenesis *in vitro*. Representative SEM images of hBMSCs cultured on CSC (**A**), MCSC 1:7 (**B**) and MCSC 1:3 (**C**) scaffolds for 3d. (**D**) The cell proliferation of hBMSCs and the osteogenic gene expression of (**E**) COL 1, (**F**) Runx2, (**G**) OCN and (**H**) ALP) at mRNA level in hBMSCs cultured on CSC, MCSC 1:7 and MCSC 1:3 scaffolds for 14 days. (**I**) The expression of BMP-2, p-Smad1/5, and Runx2 at the protein level in hBMSCs cultured on CSC, MCSC 1:7 and MCSC 1:3 scaffolds for 14 days. ^#^p < 0.05

Next, we detected whether MCSC scaffolds were appropriate for bone regeneration. The attachment and morphology of hBMSCs cultured on CSC, MCSC 1:7 and MCSC 1:3 scaffolds were observed by SEM. Three days after incubation, the hBMSCs were seen to be attached on the surface of the pore struts and well-distributed (Fig. 8A–C). Then the cell proliferation of cultured hBMSCs on scaffolds was determined by CCK-8. As shown in Fig. 8D, all tested scaffolds promoted hBMSCs proliferation, and the MCSC 1:7 and MCSC 1:3 scaffolds had significantly higher efficacy than CSC scaffolds at day 1 and day 7 in promoting cell proliferation. Notably, hBMSCs cultured on MCSC 1:3 scaffolds had the highest proliferation rate at all time points (Fig. 8D). Moreover, the expression of osteogenic genes was significantly higher in cells cultured on MCSC 1:3 scaffolds than that on MCSC 1:7 and CSC scaffolds (Fig. 8E–H). Compared with CSC scaffolds, the expression of bone morphogenetic protein (BMP)-2, phosphorylated Smad1/5 and Runx2 at the protein level was remarkably upregulated in hBMSCs cultured on the MCSC 1:7 and MCSC 1:3 scaffolds (Fig. 8I), indicating BMP/Smad signaling was, at least in part, involved in promoting osteogenesis.

#### Bone regeneration *in vivo*

**Figure 9:**
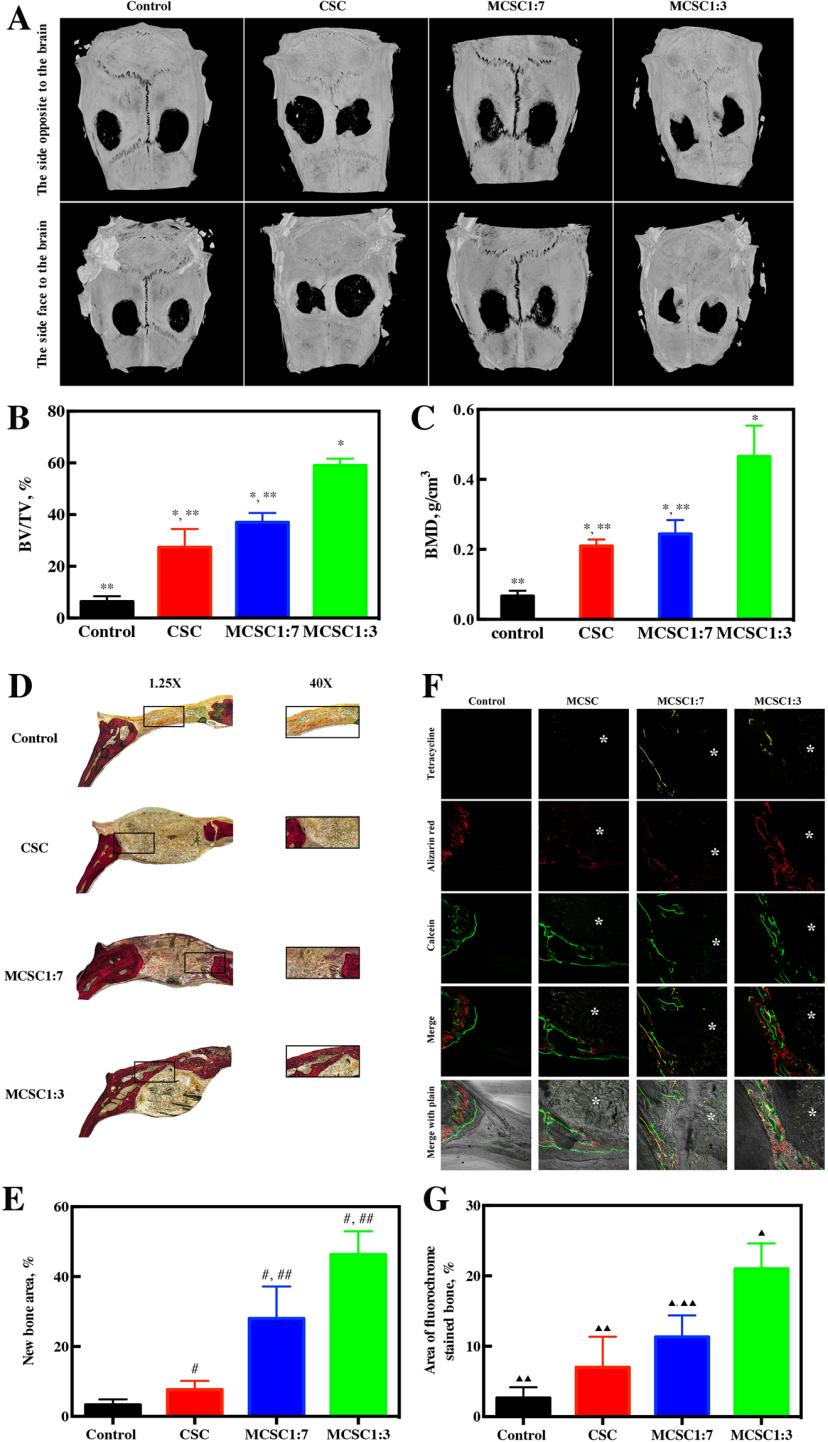
The hBMSCs seeded scaffolds promoted osteogenesis *in vivo*. (**A**) Representative Micro-CT images of bone defects. (**B**) BV/TV ratio and (**C**) BMD, *p < 0.05 versus control group, **p < 0.05 versus MCSC 1:3 scaffolds group. (**D**) Representative Van Gieson’s picrofuchsin staining images. The new bone appears red. (**E**) Statistical data of new bones, ^#^p < 0.05 versus control group, ^##^p < 0.05 versus CSC. (**F**) Row 1 (yellow) represents tetracycline at week 2, row 2 (red) represents alizarin red at week 4, row 3 (green) represents calcein at week 6, row 4 represents merged images of three fluorochromes for the same group and row 5 represents merged images from a plain microscope. *represents implanted scaffolds material. (**G**) The percentage of each fluorochrome area. ^▲^p < 0.05 versus control group, ^▲▲^p < 0.05 versus MCSC1:3 scaffolds group. BMD, bone mineral density; BV/TV, bone volume per tissue volume.

To validate the osteogenesis effects of magnetic scaffolds, a bone defect rat model was established. Compared with rats in the control group, rats treated with CSC, MCSC 1:7 and MCSC 1:3 scaffolds showed obvious signs of bone formation and decreased defect area as indicated by micro-CT scanning (Fig. 9A). Moreover, bone formation in rats treated with MCSC 1:3 scaffolds was more impressive compared to that in rats treated with MCSC 1:7, and CSC scaffolds (Fig. 9A). The trabecular bone parameters, such as bone mineral density (BMD) and bone volume per tissue volume (BV/TV) were also measured. The BV/TV in rats treated with MCSC 1:3 scaffolds was 57.32 ± 3.53% which is significantly higher than that of the MCSC 1:7 scaffolds group (36.54 ± 2.08%), CSC scaffolds group (27.63 ± 4.09%) and control group (6.33 ± 1.2%) (Fig. 9B). Additionally, the BMD in rats treated with MCSC 1:3 scaffolds was significantly higher than that in rats treated with other scaffolds (Fig. 9C). Furthermore, Van Gieson’s picrofuchsin staining showed that MCSC 1:3 and MCSC 1:7 scaffolds both exhibited osteogenic induction ability, and the effects MCSC 1:3 was more prominent than the latter (Fig. 9D). Additionally, the histomorphometric assay showed that the percentage of new bone area in MCSC 1:3 scaffolds and MCSC 1:7 scaffolds groups was significantly higher than that in CSC scaffolds and control groups (Fig. 9E). Bone formation and mineralization were also determined by calcein fluorescence assay. The fluorescence signaling located near the scaffolds indicated the new bone formation of the loci around the scaffolds (Fig. 9F). Notably, the fluorescence signaling in the MCSC 1:3 scaffolds group was higher over other scaffolds (Fig. 9F,G). These data demonstrate that the MCSC 1:3 scaffolds could effectively promote bone formation *in vivo*.

## Discussion

Surgical resection of osteosarcoma may cause bone defects, and residual tumor tissues induce tumor recurrence^30^. The fabrication of multifunctional scaffolds with potent anti-tumor activity and osteogenesis property is a critical and promising strategy for treatment of the tumor-related bone defects. Herein, we, for the first time, fabricated the MCSC scaffolds combining the ability of enhanced new bone regeneration with the excellent capacity of chemo-photothermal synergetic therapy against osteosarcoma (Fig. 1).

The multifunctional MCSC porous scaffolds were fabricated by a freeze-drying method using SrFe_12_O_19_, CaSiO_3_ and CS as original materials. The pore sizes of interconnected macropores were mainly distributed around 100~300 μm, which were formed due to the distillation of ice crystals during the freeze-drying process (Fig. 3)^31^. These macropores not only supported the adhesion and spreading of hBMSCs (Fig. 8), but also promoted the ingrowth of newly formed bone tissues (Fig. 9). The CS and mesporous CaSiO_3_ microspheres played a vital role in cell performance and bone regeneration, too. The CSC scaffolds without magnetic particles possessed excellent cytocompatibility, and exhibited better bioactivity than blank control group (Fig. 9). On one hand, CS was an important osteoconductive matrix due to its excellent biocompatibility and biodegradability^32^; on the other hand the Ca and Si from CaSiO_3_ particles might have the potential to improve the stem cell viability and osteogenic differentiation^33,34^.

In order to eradicate the residual tumor cells to avoid tumor recurrence, we employed the strategies of drug delivery system and photothermal therapy. It was noted that NIR promoted the DOX release from MCSC scaffolds and the cumulative release ratio is 79.3% at 24 h, indicating anti-tumor drugs could be rapidly released locally to combat malignant cells and could potentially minimize the side effects caused by chemo-regimens. This phenomenon could possibly due to accelerated Brownian movement and the accelerated degradation of the chitosan matrix due to the increased temperatures in the particle surrounding, as suggested by a previous study^21^. In addition, DOX could also be gradually released from scaffolds at relatively low ratio, one potential translational usage of this property is that other potent anti-tumor drugs or drug combination could be carried to designated area and released at optimal ratio to combat tumors. Compared with MCSC, MCSC/DOX induced a higher cell death ratio and irradiation further enhanced their anti-tumor effects in a dose dependent manner. When irradiated twice, MG-63 cells treated with MCSC 1:3/DOX exhibited pronounced cell viability decrease compared with MCSC 1:7/DOX. This could be elucidated by that the temperature elevation in MCSC 1:3/DOX scaffolds was higher than that in MCSC 1:7/DOX scaffolds and the amount of DOX released by MCSC 1:3/DOX scaffolds was more than that of MCSC 1:7/DOX scaffolds (Fig. 5A–C). *In vivo* analyses further proved that NIR significantly promoted the temperature in tumors treated with MCSC1:3 scaffolds and the tumor volume was significantly smaller than that treated with MCSC1:3 scaffolds alone. Xenograft mouse model validated these results, which showed that NIR synergized with MCSC1:3 scaffolds to exert proliferative arrest effects and NIR enhanced the anti-tumor effects of MCSC1:3/DOX scaffolds. These findings strongly suggest that the MCSC1:3/DOX scaffolds had potent anti-tumor effects. However, the establishment of *in situ* bone metastasis animal model is very difficult and the xenograft mouse model of bone cancer could reflect the anti-tumor effects of PTT synergy with MCSC scaffolds as indicated by studies in solid tumors^35–37^. We also admit that the fact that NIR could be more difficult in reaching bone tissues. In future studies, we will use a more suitable mouse model to test the NIR penetration in bone tissues and its combination with MCSC scaffolds in combating malignant cells in bones. Interestingly, in the present study, we could see that the more loading of magnetic field, the higher anti-tumor effects. The phenomenon could possibly be explained by the fact that PTT induced a higher local temperature in tumor cells, thereby increasing the expression of reactive oxygen species (ROS) and heat shock proteins^38^ as well as decreasing concentrations of metabolites associated with cell proliferation and tumor growth^39^. Further studies are needed to explore the mechanism of PTT synergy with MCSC scaffolds in inhibiting tumor cell proliferation.

The CSC could support the adhesion, spreading and proliferation of hBMSCs (Fig. 8), but its property for enhancing new bone regeneration was limited (Fig. 9). Recently, It was reported that strong static magnetic field (SMF) not only up-regulated the stem cell differentiation, but also stimulated ectopic bone formation^40^. In this study, we incorporated SrFe_12_O_19_ ferrite particles into the CSC scaffolds, resulting in the formation of MCSC porous scaffolds. COL1, OCN, Runx2 and ALP are essential for osteogenesis and their upregulation is a positive indicator for osteogenesis^41,42^. Interestingly, the MCSC scaffolds significantly augmented the expression of these indicators for hBMSCs, suggesting MCSC scaffolds have a potential in promoting bone formation. *In vivo* analysis revealed that the markers for bone formation such as BV/TV and BMD were increased. Micro-CT scan, Van Gieson’s picrofuchsin staining and calcein fluorescence assay further proved that hBMSCs loaded MCSC scaffolds could effectively promote osteogenesis. The role of BMP/Smad signaling pathway in bone formation has been widely acknowledged in bone formation^43,44^ and Runx2 has been reported to the downstream mediator for Smad1/5 ^45^. The present study revealed that the expression of BMP, Smad1/5 and Runx2 was upregulated in hBMSCs loaded MCSC scaffolds. These data indicate that BMP/Smad/Runx2 signaling was, at least in part, involved in the magnetic scaffolds promoted osteogenic differentiation in hBMSCs. Furthermore, we observed that the higher ratio of ferrite particles, the higher expression of osteogenic proteins and bone formation. It was reported that composites containing SrFe_12_O_19_ can potentially better facilitate bone formation^46^, which was in line with this study. Therefore, we could reasonably infer that M-type ferrite particles, at least SrFe_12_O_19_, had the potential to contribute to osteogenesis, and more studies are warranted to unveil the underlying mechanism.

## Conclusions

This study suggested that the MCSC scaffolds could load anti-tumor drugs and synergy with PTT to combat tumor cells. Additionally, MCSC scaffolds could enhance the proliferation of hBMSCs and promote bone formation. These results strongly suggest that MCSC scaffolds are bi-functional and may have a potential role in patients with surgical resection of malignant bone tumors treatment. In summary, we have derived pre-clinical data supporting a role for MCSC scaffolds in harbouring potent anti-tumor and bone regeneration capability both *in vitro* and *in vivo*. Nevertheless, more studies are needed to evaluate the efficacy and application of MCSC in clinics for patients with bone defects due to bone tumor resection.

## Methods

### Preparation of magnetic calcium silicate/chitosan porous (MCSC) scaffolds

Mesoporous calcium silicate microspheres were prepared by using organic template method. In brief, cetyltrimethyl ammonium bromide (CTAB, 1.76 g) and ammonium hydroxide (NH_3_·H_2_O, 3.20 ml) were dissolved in deionized water followed by stirring for 30 min. Under vigorous agitation, tetraethyl orthosilicate (TEOS, 9.33 ml) and 9.73 g calcium nitrate tetrahydrate were successively dissolved in the above solution, and were further aged for 20 h. The precipitates (mesoporous calcium silicate microspheres) were washed by deionized water, dried at 80 °C and calcined at 550 °C for 4 h.

M-type ferrite (SrFe_12_O_19_) particles with approximately 0.5 μm were obtained from Hengdian Group DMEGC Magnetics Co., Ltd. The ferrite powders (0.25 or 0.125 g) and mesoporous calcium silicate microspheres (0.75 or 0.875 g) were added into CS solutions (4.00 wt%, 25.00 ml). The mixtures were injected in the cylindrical mold with a diameter of 15.6 mm and height of 19.0 mm. Under external strong magnetic fields, the samples were frozen at −20 °C in a deep freezer (DW-25L262, Haier, Qingdao, China). The solidified mixtures were freeze-dried at −35 to −50 °C for 48 h by a in a freeze-drying machine (LGJ-10D, Four-Ring Science Instrument Plant Beijing Co., Ltd, Beijing, China). The MCSC scaffolds were neutralized by a NaOH solution (10.0 wt%) for 24 h, and then washed with deionized water. Finally, the MCSC scaffolds were magnetized by a magnetization machine (ME-1520, Magele Technology CO., Ltd, Shanghai, China), resulting in the same magnetic domain directions and high magnetic fields. The mesoporous calcium silicate/CS (CSC) scaffolds which served as control group were fabricated under the same condition without ferrite powders. The MCSC scaffolds with the ferrite/calcium silicate mass ratio at 1:7 and 1:3 were named as MCSC 1:7 and MCSC 1:3, respectively.

### Scaffold characterization

The morphology of CSC and MCSC scaffolds (diameter: 8 mm, height: 4 mm) were characterized by field-emission scanning electron microscopy (FESEM) with energy-dispersive spertrometry (EDS, JSM-6380LV, JEOL, Tokyo, Japan). The porous structures of mesoporous calcium silicate microspheres were characterized by transmission electron microscopy (TEM, JEOL2100, JEOL, Tokyo, Japan). N2 adsorption-desorption isotherms of mesoporous BG microspheres of mesoporous microspheres were determined by automatic surface area and porosity analyzer (AUTOSORB-1-C, Quantachrome, Tallahassee, Florida, USA) at 77 K. The crystalline structure of the scaffolds was determined by X-ray power diffraction (XRD; D/MAX-III C, Tokyo, Japan), and the functional groups were analyzed by Fourier transform infrared (FTIR; Nicolet 5DX, Thermo Fisher Scientific, Massachusetts, USA). The magnetic properties of magnetic scaffolds were analyzed by vibrating sample magnetometer (VSM, Changchun City Yingpu Magnetic Technology Development Co., LTD, Jilin, China).

### Drug loading

A DOX solution with a concentration of 3.75 mg/l was prepared by dissolving DOX in distilled water. The DOX loading were carried out by the injection of 200 μl DOX solution in scaffolds, followed by freeze-drying in a freeze-drier machine (LGJ-10D, Four-Ring Science Instrument Plant Beijing Co., Ltd, Beijing, China). The CSC, MCSC 1:3 and MCSC 1:7 porous scaffolds loaded with DOX were denoted as CSC/DOX, MCSC 1:3/DOX and MCSC 1:7/DOX, respectively.

### NIR laser-induced temperature increase and *in vitro* drug release test

A PBS solution with pH value of 7.4 was prepared by adding 5.8032 g Na_2_HPO_4_·12H_2_O and 0.8894 g NaH_2_PO_4_·2H_2_O into 1000 mL distilled water. A NIR laser was fixed 15 cm higher than the center of the scaffolds. Under the irradiation of NIR (λ = 808 nm, 4.6 W/cm^2^), the temperatures of the media with the scaffolds were detected by thermocouple thermometer (Brannan, Cleator Moor, England). The NIR-light-triggered drug release tests of MCSC 1:3/DOX and MCSC 1:7/DOX scaffolds were performed by the immersion of DOX loaded scaffold in 5 ml PBS solution under orbital shaking of 80 rpm. In brief, the probe of NIR laser (λ = 808 nm, 4.6 W·cm^−2^) was fixed 15 cm higher from the center of the scaffolds. At given time intervals, the scaffolds were irradiated by NIR laser for 6 min. Then 1.0 ml DOX-release medium was extracted and supplemented with 1.0 ml fresh PBS. The DOX concentrations of the DOX-release medium were characterized by fluorescence spectrophotometer (F-4600, Hitachi, Tokyo, Japan) using a xenon lamp as excitation source (λ = 495 nm). The *in vitro* drug release of the MCSC 1:7/DOX or MCSC 1:3/DOX scaffolds without NIR irradiation was performed with similar procedures.

### Cell Culture

The human MG-63 and MNNG osteosarcoma cell line was obtained from ATCC, cultured with high glucose DMEM (Gibco) containing 10% FBS (Gibco) and 1/100 penicillin-streptomycin (Gibco). All cells were kept in humidified atmosphere containing 5% CO_2_ at 37 °C and subject for anti-tumor analyses.

### Cell viability assay

A number of 1 ×10^6^ MG-63 cells were incubated with MCSC 1:7, MCSC 1:7/DOX, MCSC 1:3 or MCSC 1:3/DOX scaffolds for 24 h, then cells were irradiated by NIR laser with a power density of (λ = 808 nm, 4.6 W/cm^2^) for 3 min or 6 min and cultured for another 24 h. Cells were without irradiation were served as control. Then cell viability was evaluated by cell counting kit-8 (CCK-8, Beyotime, China) according to manufacturer’s instructions.

As for irradiation twice, the MG-63 cells were first irradiated by NIR for 6 min post 24 h treatment of MCSC 1:7, MCSC 1:7/DOX, MCSC 1:3 or MCSC 1:3/DOX scaffolds, cultured for another 24 h and cells were irradiated the second time for 6 min. The size of MCSC scaffolds for all *in vitro* analyses was 15 mm × 2 mm and the amount of the DOX was 150 ng.

### Live/dead assay

A number of 2×10^6^ MG-63 cells were seeded in six-well-plate, 24 h later, cells were incubated with MCSC scaffolds, MCSC/DOX scaffolds for another 24 h with or without 6 min’s NIR irradiation (λ = 808 nm, 4.6 W/cm^2^, irradiated post scaffolds incubation for 24 h). The live and dead cells were analyzed according to manufacturer’s instruction (Tecan, Switzerland) and observed under an inverted fluorescence microscope (Olympus, Japan).

### EGFP transfection

Approximately 1 × 10^6^ MNNG cells were seeded in a six well plate and cultured for 24 h. Then lentivirus containing EGFP at a multiplicity of infection (MOI) of 150 plaque-forming units (PFU) (Genechem, Shanghai, China) were incubated with MNNG cells. The next day, the culture medium was replaced with fresh medium and the fluorescence density was evaluated under inverted fluorescence microscope (Olympus, Tokyo, Japan).

### PTT in tumors *in vivo*

The xenograft mouse model bearing osteosarcoma cells was constructed as previously described^33^. In brief, the osteosarcoma MNNG (5 × 10^6^) cells with or without EGFR transfection were subcutaneously inoculated into the left flank of nude mice (4–6 weeks old, 20 ± 2 g). When tumors were palpable, the mice were randomly divided into MCSC 1:3 scaffolds, MCSC 1:3/DOX scaffolds with laser irradiation, MCSC 1:3 scaffolds and MCSC 1:3/DOX scaffolds without irradiation groups (n = 5/group). A skin incision was made at the edge of the tumor and then the scaffold (diameter: 4 mm, height: 2 mm) was implanted.

For laser irradiation, the mice were exposed to the NIR laser (0.3 W/cm^2^) for 6 min post scaffolds implantation. Meanwhile, the temperature in the tumor was monitored by real-time IR thermal imaging system. The first NIR treatment time was regarded as day 0. From day 0, tumor sizes were measured with calipers and their volumes were calculated by the following the formula: (length × width^2^)/2. V_0_ is the initial tumor volume-scaffold volume at day 0. The relative tumor size = V/V_0_. Whole-body fluorescent imaging was performed at day 0 and day 12. The mice were sacrificed at the 12^th^ day, and their tumors were immersed in 4% neutral buffered formalin for 24 h, then the tissues were embedded in paraffin and cut to 5 μM sections. Afterwards, the sections were dehydrated with gradient alcohol and xylene, then they were subjected for hematoxylin-eosin (H&E) staining according to manufacturer’s instructions (Beyotime, China).

The tumor cell necrosis rate was also measured. Five slices were selected, and five random visions were measured. Tumor cell necrosis rate (TCNR) was measured according to the formula, TCNR = the number of necrotic cells/the total cell number × 100%. The size of MCSC scaffolds for all *in vitro* analyses was 6 mm X 2 mm and the amount of the DOX was 300 ng.

### Isolation of hBMSCs

hBMSCs were harvested from healthy donors as previously described^47^. hBMSCs were cultured in α-MEM (Sigma-Aldrich, St. Louis, MO, USA), supplemented with 10% fetal bovine serum (FBS, Gibco, USA) and 1/100 penicillin-streptomycin (Gibco), cells were maintained in humidified atmosphere containing 5% CO_2_ at 37 °C and hBMSCs at 3–5 passages were used for bone regeneration analysis.

### Morphology assay

A number of 1.0 × 10^4^ hBMSCs at its third passage were seeded in a 24-well culture plate and incubated scaffolds. To evaluate the cell attachment and morphology in CSC, MCSC 1:7 and MCSC 1:3 scaffolds at 1, 3 and 7 days, the scaffolds were fixed in 2.5% glutaraldehyde for 20 min, washed with PBS for three times, dehydrated in a graded ethanol and hexamethyldisilizane. Then dehydrated cellular scaffolds were coated with gold and observed by SEM (Hitachi, S-4700, Tokyo, Japan).

### Real-time polymerase chain reaction (RT-PCR)

After hBMSCs were treated with the scaffolds for 14 days, then total RNA was extracted using the TRIzol reagent (Invitrogen, #15596-026). cDNA was reverse-transcribed using SuperScript First-Strand Synthesis System (Invitrogen, #1209992, USA). The expression of ALP, Runx2, OCN and COL1 was measured by RT-PCR using FastStart Universal SYBR Green Master (Roche, #04913914001, USA) in a ViiA 7 Real-Time PCR System (Applied Biosystems, USA). The procedure for this reaction is 95 °C, 5 min; 34 cycles for 95 °C, 5 min and 60 °C, 40 s; and 72 °C, 5 min. The data was analyzed by 2^−ΔΔCt^ method and actin was used as the reference gene. The primers for RT-PCR are listed in Table 1.

**Table 1:** The primers for RT-PCR.

	Forward (5′-3′)	Reverse (5′-3′)
COL1	GGAGACGGCTATTTTGGGACG	TCCTTGAGTGGAGCTTCCATT
Runx2	CCGAGACCAACCGAGTCATTTA	AAGAGGCTGTTTGACGCCAT
OCN	TCAACAATGGACTTGGAGCCC	AGCTCGTCACAATTGGGGTT
ALP	CAAGGATGCTGGGAAGTCCG	CTCTGGGCGCATCTCATTGT
β-actin	GTCATCCATGGCGAACTGGT	CGTCATCCATGGCGAACTGG

### Western blot analysis

The hBMSCs were treated with scaffolds for 14 days, then the Cells were lysed in lysis buffer supplemented with protease and phosphatase inhibitor (Thermo Scientific, #78440, USA). Protein concentration was assessed by BCA (Cell Signalling Technology, USA). Equal amount of protein (20 μg/lane) was separated by 8–12% SDS-PAGE. Then protein was transferred to PVDF membrane (Millipore, USA), the non-specific signals were blocked with 5% (w/v) skim-milk at room temperature for 1 h. Afterwards, primary antibodies against GAPDH (1:1000, Cell Signaling Technology, USA), BMP-2(1:1000, Cell Signaling Technology, USA), p-Smad1/5 (1:1000, Cell Signaling Technology, USA) and Runx2 (1:1000, Cell Signaling Technology, USA) were incubated overnight at 4 °C. Horseradish peroxidase (HRP)-conjugated polyclonal goat secondary antibodies were then incubated for 2 h at room temperature. The protein bands were detected by electrochemiluminescence (ECL) method (Thermo Scientific, #34095). GAPDH was served as internal reference.

### Bilateral critical-sized calvarial-defect rat model

Twenty Sprague-Dawley male rats (300–350 g) obtained from Silaike (Shanghai, China) were used to establish the bilateral critical-sized calvarial-defect rat model (n = 5/group). Two parietal defects were created (diameter: 5 mm, height: 2 mm), then either CSC, MCSC 1:7 and MCSC 1:3 scaffolds were implanted into the defect and the incisions were closed. A polychrome sequential fluorescent labeling method was performed to observe bone formation and mineralization on rats at week 12 post-treatment. At 2, 4 and 6 weeks after implantation, fluorochromes [25 mg/kg tetracycline (TE; Sigma, USA), 30 mg/kg alizarin reds (AL; Sigma, USA) and 20 mg kg 1 calcein (CA; Sigma, USA)] were injected (i.p.) to rats. At 12 weeks post- implantation, the rats were sacrificed and micro-CT (Skyscan 1176, Kontich, Belgium). Three dimention (3-D) images were reconstructed using the 3-D Creator software. Besides bone volume to total bone volume (BV/TV) and local bone mineral densities (BMD).

After dehydration in ascending concentrations of alcohol the undecalcified specimens were embedded in poly-methylmethacrylate and 150 mm thick sections in the orientation of the sagittal surface were obtained using a microtome (Leica, Hamburg, Germany). The sections were observed for fluorescent labeling using a confocal microscope (Leica, Heidel-berg, Germany). The excitation/emission wavelengths of the chelating fluorochromes used were 405/560–590 nm (tetracycline, yellow), 543/580–670 nm (alizarin red, red) and 488/500–550 nm (calcein, green). Histological analyses were carried out by Van Gieson’s picrofuchsin staining. With a computer-based image analysis system (Image-Pro Plus 6.0, Media Cybernetics, Silver Springs, MD), the area of new bone was quantified as a percentage of the total bone defect area.

### Statistical analysis

All statistical analyses were performed using SPSS 20.0 (SPSS Inc., Chicago, IL, USA). All the data were expressed as means ± standard deviation (SD) and were analyzed using one-way ANOVA with a post hoc test. A two-tailed p-value < 0.05 was considered statistically significant.
